# An Assessment of Mental Health Outcomes During the COVID-19 Pandemic

**DOI:** 10.1007/s10597-021-00876-9

**Published:** 2021-07-20

**Authors:** Edward Magalhaes, Alexis Stoner, Joshua Palmer, Robert Schranze, Savannah Grandy, Shilpa Amin, Ning Cheng

**Affiliations:** 1 Edward Via College of Osteopathic Medicine, Virginia Campus, 2265 Kraft Dr, Blacksburg, VA 24060 USA; 2 Edward Via College of Osteopathic Medicine, Carolinas Campus, 350 Howard St, Spartanburg, SC 29303 USA; 3 Edward Via College of Osteopathic Medicine, Auburn Campus, 910 South Donahue Dr, Auburn, AL 36832 USA

**Keywords:** COVID-19, Pandemic, Mental health, Depression, Stress, Anxiety

## Abstract

In late 2019, the first case of COVID-19 was reported in Wuhan, China. Soon after, cases began to spread globally. This study aimed to examine the psychological impact of the COVID-19 pandemic on the adult population in the United States. We conducted an exploratory cross-sectional study using an anonymous online survey methodology distributed to participants across 13 states. The data collected included demographical information and outcomes from validated mental health screeners (GAD-7, PHQ-9, and IES-R) to assess levels of anxiety, depression, and stress. A total of 1356 participants completed the survey. GAD-7, PHQ-9, and IES-R levels differed significantly (p < 0.05) according to age, gender, and educational level. There was also significant difference between GAD-7 level as well as IES-R level between healthcare and non-healthcare workers (p = 0.02 and p = 0.028). Overall, this study has helped to garner a better understanding of COVID-19’s impact on mental health outcomes.

## Introduction

December 2019, a cluster of pneumonia cases were reported in Wuhan, China and a novel Coronavirus was eventually identified. By early March of 2020, travel restrictions were put in place, the World Health Organization (WHO) declared the novel coronavirus outbreak to be a pandemic, and the President of the United States (US) declared a national emergency (World Health Organization, 2020). It was during this time that structure and schedules were completely disrupted, and community members were faced with isolation in their homes and the closure of public services began.

In the US, the COVID-19 pandemic led to the recommendation and implementation of social distancing and isolation as approaches to curb the spread of the virus. As a result, many communities in the US had to quickly adapt their habits of social interaction to an online and virtual environment. Additionally, many families had to also adjust their daily routines, rituals, roles, and responsibilities at home. The literature has shown that sudden changes in work and home dynamics can increase the risk of the following distress reactions: changes in sleep, decreased sense of safety, physical symptoms, irritability, distraction, avoidance, and isolation (Ursano et al., 2017).

Throughout history, there have also been studies examining the psychosocial wellbeing of society after natural disasters and public health outbreaks, such as the Spanish Flu in 1918, the Asian Flu in 1957, SARS in 2002 and 2003, the H1N1 Influenza pandemic of 2009, and the Ebola virus outbreak in 2013 (Douglas et al., 2009; Zhang et al., 2020). These studies have shown that healthcare workers are often at increased risk for stress and anxiety due to unclear and changing policies, illness stigma, and altered home and work boundaries during a pandemic (Morganstein et al., 2017). In addition, some other at-risk populations such as adolescents, parents, caregivers, and the economically disadvantaged, who depend on external systems of care for social support, food, and structure in their daily lives (Griffith & Kohrt., 2016) can be psychologically affected in the setting of disasters and pandemics. Studies have also shown that these disturbances can lead to increased levels of depression, anxiety, and post-traumatic stress disorder (PTSD) in these populations post-disaster. (Douglas et al., 2009; Kang et al., 2020).

As the coronavirus continued to sweep across the world a group of researchers in Wuhan, China conducted a study on the effects of COVID-19 on healthcare workers. Their study found that a considerable proportion of healthcare workers reported increased depression, anxiety, insomnia, and stress during the peak of the COVID-19 pandemic in China (Lai et al., 2020). However, their study did not examine COVID-19’s impact on the surrounding non-healthcare working community.

The goal of this study is to expand on the known literature and the Wuhan study to help better understand COVID-19’s impact on the mental wellbeing of healthcare and non-healthcare adult community members. An additional goal of this study is to see what impact telecommunications have on mental wellbeing during this global pandemic.

## Methods

For the purposes of this study we utilized an exploratory cross-sectional study using a survey methodology approach, which was employed to help better understand the impact of the COVID-19 pandemic on mental health outcomes. This study was approved by the Edward Via College of Osteopathic Medicine’s (VCOM) Institutional Review Board.

The survey was an anonymous tool developed using validated mental health screeners and basic demographic information. It included the 7-item Generalized Anxiety Disorder scale (GAD-7) to assess anxiety, the 9-item Patient Health Questionnaire (PHQ-9) to assess depression, and the 22-item Impact of Event Scale Revised (IES-R) to assess stress. The GAD-7 is a self-reporting questionnaire for screening and measuring severity of generalized anxiety disorder and it was chosen to address the research questions about anxiety levels in the setting of COVID. The PHQ-9 is a screener for the presence and severity of depression and was chosen to gauge depressive symptoms in the setting of COVID. GAD-7 and PHQ-9 are both free, reliable, and established screening tools. The IES-R is a self-reporting measure that assesses subjective distress caused by traumatic events and was used to assess how people were affected by COVID. The revised version was used over the original because it includes seven additional questions about hyperarousal. All three of these questionnaires were adjusted to ask about experiencing changes over the last month rather than two weeks to better assess any changes experienced during quarantine. Demographic variables included age, gender, ethnicity, health care provider status and role, current employment status, state of residence and region, income, highest level of education, and marital status.

Our study was initially looking at the Commonwealth of Virginia, but after additional interest we expanded our study to South Carolina, and then to an additional 11 states that we were able to establish point of contacts. Therefore, our exploratory survey became state-stratified and was distributed to adults, 18 years of age or older, throughout 13 states that we categorized into 3 regions: North Region (Massachusetts, New York, New Jersey, Pennsylvania, Ohio), South Region (Virginia, North Carolina, South Carolina, Florida, Alabama, Louisiana), and West Region (California and Washington). Data collection occurred for a span of one month in each state. The Commonwealth of Virginia was surveyed during the month of May 2020, South Carolina was then surveyed during the month of June 2020, and all remaining states were surveyed during the month of August 2020.

Due to COVID-19 restrictions we employed a digital recruitment strategy that was completed via points of contact in the respective states in order to eliminate the need for in-person contact during periods of quarantine or stay at home orders. Distribution was employed using VCOM’s network of clinical sites, hospital systems, various medical associations, and community health and outreach organizations. We also utilized social media platforms to increase the geographic reach of the study, by capitalizing on increased social media attention with people working from home.

Results of the study were analyzed using SPSS. Descriptive statistics were used to analyze the characteristics of survey participants. Chi-square tests (Fisher’s exact tests for samples less than 10) were used to assess the differences between groups. A significance level of α = 0.05 was used to demonstrate a difference between the samples.

We also conducted multinomial logistic regression models of the GAD-7, PHQ-9, and IES-R respectively. We selected the most representative variables for our multinomial logistic regression models. The covariates we included in the models were, age, gender, ethnicity, health care provider, marital status, education, and income.

All authors of this study certify our responsibility in accepting our conduct of the study and for the analysis and interpretation of the data. We certify our responsibility that we helped write the manuscript and agree with the decisions about it.

## Results

### Demographics

A total of 1356 participants completed the survey. A majority of them were between the ages of 35–55 (54.9%), identified as female (84.1%), and White/Non-Hispanic (89.8%) (Table 1). Most of the respondents (79.2%) were employed and 59.1% had a bachelor’s or master’s degree, while 33.6% of respondents identified as a healthcare worker (Table 1). About half of our sample were from states located in the South Region (55.6%). Variability was found across education and income levels (Table 1).

**Table 1:** Sample demographics

Variable	n (%)
Age	
18–34	357 (26.3)
35–55	744 (54.9)
55+	255 (18.8)
Gender	
Male	214 (15.8)
Female	1141 (84.1)
Other	1 (0.1)
Ethnicity	
White/Non-Hispanic	1217 (89.8)
Black/African-American	49 (3.6)
Other	89 (6.6)
Healthcare provider role	
Doctor	121 (26.5)
Nurse	117 (25.7)
Other	218 (47.8)
Total	456 (33.6)
Employment status	
Unemployed	124 (14.2)
Employed	710 (79.2)
Retired	63 (7.0)
Missing	453 (33.4)
Marital status	
Single/never married	277 (20.4)
Married/domestic partnership	894 (66.0)
Divorced/separated/widowed	183 (13.5)
Missing	2
Region	
North^a^	570 (42.0)
South^b^	754 (55.6)
West^c^	32 (2.4)
Education	
Associate and under	337 (24.9)
Bachelors/masters	802 (59.1)
Professional degree	217 (16.0)
Income	
$0–$49,999	450 (33.8)
$50,000–$99,999	534 (40.1)
$100,000+	348 (26.1)
Missing	24 (1.77)

### PHQ-9

There was a significant difference between the level of depression reported among healthcare and non-healthcare workers (p = 0.02). Of participants with a mild/moderate PHQ-9 score, 67.4% were non-healthcare workers and of participants with a severe PHQ-9 score, 74.4% were non-healthcare workers (Table 2). Additionally, level of reported depression significantly differed (p = 0.00) among types of healthcare workers, with over half (54.1%) of those who reported severe levels identifying as nurses (Table 2). PHQ-9 levels also differed significantly according to age, gender, marital status, education, income, telehealth utilization, and having a prior mental health disorder (Table 2). Participants who reported a moderate or severe PHQ-9 score were more likely female, married, between the ages of 35–54, employed, did not have a prior mental health diagnosis, were not using telehealth during the pandemic and had a bachelor/master’s degree (Table 2). PHQ-9 scores did not differ significantly according to one’s ethnicity, region of residence, or level of involvement in a child’s schooling (Table 2).

**Table 2:** PHQ-9 assessment results

Variable	Minimal n (%)	Moderate n (%)	Severe n (%)	p value
Healthcare employee				
Healthcare	213 (37.1)	208 (32.6)	37 (25.3)	0.02
Non-healthcare	359 (62.9)	431 (67.4)	109 (74.7)	
Previous mental health diagnosis				
No prior diagnosis	462 (81.2)	398 (63.1)	70 (49.0)	0.00
Previous diagnosis	107 (18.8)	233 (36.9)	73 (51.0)	
Age				
18–34	130 (22.8)	176 (27.5)	52 (35.6)	0.00
35–54	306 (53.6)	361 (56.4)	77 (52.7)	
55+	135 (23.6)	103 (16.1)	17 (11.6)	
Gender				
Male	116 (20.3)	82 (12.8)	16 (11.0)	0.01*
Female	455 (79.7)	557 (87.0)	130 (89.0)	
Other	0 (0.0)	1 (0.2)	0 (0.0)	
Ethnicity				
White/Non-Hispanic	508 (89.1)	577 (90.2)	133 (91.1)	0.82*
Black/African-American	24 (4.2)	22 (3.4)	3 (2.1)	
Other	38 (6.7)	41 (6.4)	10 (6.8)	
Healthcare provider role				
Doctor	65 (31)	52 (24.9)	4 (10.8)	0.00*
Nurse	38 (18.1)	59 (28.2)	20 (54.1)	
Other	107 (51.0)	98 (46.9)	13 (35.1)	
Employment status				
Employed	282 (78.8)	344 (80.0)	84 (77.1)	0.03*
Unemployed	41 (11.5)	62 (14.4)	21 (19.3)	
Retired	35 (9.8)	24 (5.6)	4 (3.7)	
Marital status				
Single/never married	93 (16.3)	139 (21.8)	45 (31.0)	0.00
Married/domestic partnership	407 (71.4)	408 (63.8)	79 (54.5)	
Divorced/separated/widowed	70 (12.3)	92 (14.4)	21 (14.5)	
Region				
North^a^	232 (40.6)	268 (41.9)	70 (47.9)	0.17*
South^b^	320 (56.0)	362 (56.6)	73 (50.0)	
West^c^	19 (3.3)	10 (1.6)	3 (2.1)	
Education				
Associate and under	120 (21.1)	164 (25.6)	53 (36.3)	0.00
Bachelor/masters	333 (58.4)	387 (60.5)	82 (56.2)	
Professional degree	117 (20.5)	89 (13.9)	11 (7.5)	
Income				
$0–49,999	155 (28.0)	235 (37.0)	60 (41.7)	0.02
$50,000–$99,999	234 (42.3)	245 (38.6)	55 (38.2)	
$100,000+	164 (29.7)	155 (24.4)	29 (20.1)	
Involvement in child’s schooling				
0–25%	93 (38)	98 (29.8)	21 (29.6)	0.16*
25–49%	37 (15.1)	40 (12.2)	6 (8.5)	
50–74%	39 (15.9)	63 (19.1)	14 (19.7)	
75–100%	76 (31.0)	128 (38.9)	30 (42.3)	
Telehealth utilization during pandemic				
Yes	167 (29.3)	253 (40.1)	59 (41.3)	0.00
No	402 (70.7)	378 (59.9)	84 (58.7)	

^a^North = Massachusetts, New York, New Jersey, Pennsylvania, Ohio

^b^South = Virginia, North Carolina, South Carolina, Florida, Alabama, Louisiana

^c^West = California, Washington

*Significance was analyzed by Fisher’s exact test. Note: Significance was determined at α = 0.05

### GAD-7

It appears that there was not a significant difference in reported GAD-7 scores between healthcare and non-healthcare workers, nor was there any between different types of healthcare provider roles. However, GAD-7 levels did differ significantly according to age, gender, employment status, education level, marital status, level of involvement in child’s schooling, and telehealth utilization during the pandemic (Table 3). Participants who reported a moderate or severe GAD-7 score were more likely female, married, between the ages of 35–54, employed, and had a bachelor/master’s degree (Table 3). GAD-7 scores did not differ significantly according to one’s ethnicity, region of residence, or level of income (Table 3).

**Table 3:** GAD-7 assessment results

Variable	Minimal n (%)	Moderate n (%)	Severe n (%)	p value
Healthcare employee				
Healthcare	168 (34.6)	245 (34.8)	44 (26.5)	0.12
Non-healthcare	317 (65.4)	460 (65.2)	122 (73.5)	
Previous mental health diagnosis				
No prior diagnosis	401 (83.0)	445 (63.8)	84 (51.5)	0.00
Previous diagnosis	82 (17.0)	252 (36.2)	79 (48.5)	
Age				
18–34	113 (23.3)	188 (26.6)	57 (34.3)	0.00
35–54	232 (47.8)	421 (59.6)	91 (54.8)	
55+	140 (28.9)	97 (13.7)	18 (10.8)	
Gender				
Male	113 (23.3)	88 (12.5)	13 (7.8)	0.00*
Female	372 (76.7)	618 (87.5)	152 (91.6)	
Other	0 (0.0)	0 (0.0)	1 (0.6)	
Ethnicity				
White/Non-Hispanic	429 (88.6)	639 (90.5)	150 (90.4)	0.70
Black/African-American	22 (4.5)	21 (3.0)	6 (3.6)	
Other	33 (6.8)	46 (6.5)	10 (6.0)	
Healthcare provider role				
Doctor	47 (28.3)	59 (24.0)	15 (34.1)	0.48
Nurse	38 (22.9)	67 (27.2)	12 (27.3)	
Other	81 (48.8)	120 (48.8)	17 (38.6)	
Employment status				
Employed	241 (76.5)	372 (80.9)	97 (79.5)	0.00*
Unemployed	39 (12.4)	60 (13.0)	25 (20.5)	
Retired	35 (11.1)	28 (6.1)	0 (0.0)	
Marital status				
Single/never married	80 (16.5)	158 (22.4)	39 (23.6)	0.05
Married/domestic partnership	329 (68.0)	455 (64.5)	110 (66.7)	
Divorced/separated/widowed	75 (15.5)	92 (13.0)	16 (9.7)	
Region				
North^a^	190 (39.2)	304 (43.1)	76 (45.8)	0.49*
South^b^	281 (57.9)	387 (54.8)	87 (52.4)	
West^c^	14 (2.9)	15 (2.1)	3 (1.8)	
Education				
Associate and under	118 (24.4)	160 (22.7)	59 (35.5)	0.01
Bachelor/masters	281 (58.1)	436 (61.8)	85 (51.2)	
Professional degree	85 (17.6)	110 (15.6)	22 (13.3)	
Income				
$0–49,999	145 (30.8)	237 (34.0)	68 (41.5)	0.08
$50,000–$99,999	189 (40.1)	289 (41.5)	56 (34.1)	
$100,000+	137 (29.1)	171 (24.5)	40 (24.4)	
Involvement in child’s schooling				
0–25%	75 (38.5)	115 (32.0)	22 (24.2)	0.04
25–49%	27 (13.8)	44 (12.3)	12 (13.2)	
50–74%	29 (14.9)	75 (20.9)	12 (13.2)	
75–100%	64 (32.8)	125 (34.8)	45 (49.5)	
Telehealth utilization during pandemic				
Yes	139 (28.8)	272 (39.0)	68 (41.7)	0.00
No	344 (71.2)	425 (61.0)	95 (58.3)	

^a^North = Massachusetts, New York, New Jersey, Pennsylvania, Ohio

^b^South = Virginia, North Carolina, South Carolina, Florida, Alabama, Louisiana

^c^West = California, Washington

*Significance was analyzed by Fisher’s exact test. Note: Significance was determined at α = 0.05

### IES-R

There was a significant difference between the IES-R level reported among healthcare and non-healthcare workers (p = 0.03). Of participants with a mild/moderate IES-R score, 66.8% were non-healthcare workers and of participants with a severe IES-R score, 75.4% were non-healthcare workers (Table 4). Reported levels of stress also significantly differed according to age, gender, education level, marital status, prior mental health diagnosis, and telehealth utilization during the pandemic (Table 4). Participants who reported a moderate or severe IES-R score were more likely female, married, between the ages of 35–54, and had a bachelor/master’s degree (Table 4). The results indicate that 65.3% of those with moderate stress and 54.6% of those with severe stress report having no previous mental health diagnosis (Table 4). Those who reported a moderate (61.6%) or severe (58.8%) IES-R level were also more likely to report not utilizing telehealth during the pandemic (p = 0.01) (Table 4). IES-R scores did not differ significantly according to one’s ethnicity, healthcare role, employment status, region of residence, level of income, or level of involvement in child’s schooling (Table 4).

**Table 4:** IES-R assessment results

Variable	Minimal n (%)	Moderate n (%)	Severe n (%)	p value
Healthcare employee				
Healthcare	141 (37.7)	287 (33.2)	29 (24.6)	0.03
Non-healthcare	233 (62.3)	577 (66.8)	89 (75.4)	
Previous mental health diagnosis				
No prior diagnosis	307 (83.0)	558 (65.3)	65 (54.6)	0.00
Previous diagnosis	63 (17.0)	296 (34.7)	54 (45.4)	
Age				
18–34	85 (22.7)	233 (27.0)	40 (33.6)	0.00
35–54	192 (51.3)	489 (56.6)	63 (52.9)	
55+	97 (25.9)	142 (16.4)	16 (13.4)	
Gender				
Male	89 (23.8)	107 (12.4)	18 (15.1)	0.00*
Female	285 (76.2)	756 (87.5)	101 (84.9)	
Other	0 (0.0)	1 (0.1)	0 (0.0)	
Ethnicity				
White/Non-Hispanic	330 (88.5)	786 (91.0)	102 (85.7)	0.31
Black/African-American	15 (4.0)	28 (3.2)	6 (5.0)	
Other	28 (7.5)	50 (5.8)	11 (9.2)	
Healthcare provider role				
Doctor	42 (30.2)	73 (25.4)	6 (20.0)	0.34
Nurse	31 (22.3)	74 (25.8)	12 (40.0)	
Other	66 (47.5)	140 (48.8)	12 (40.0)	
Employment status				
Employed	184 (79.7)	455 (78.9)	71 (79.8)	0.29*
Unemployed	29 (12.6)	79 (13.7)	16 (18.0)	
Retired	18 (7.8)	43 (7.5)	2 (2.2)	
Marital status				
Single/never married	66 (17.7)	178 (20.6)	33 (28.0)	0.00
Married/domestic partnership	258 (69.2)	569 (65.9)	67 (56.8)	
Divorced/separated/widowed	49 (13.1)	116 (13.4)	18 (15.3)	
Region				
North^a^	147 (39.3)	367 (42.5)	56 (47.1)	0.21*
South^b^	213 (57.0)	481 (55.7)	61 (51.3)	
West^c^	14 (3.7)	16 (1.9)	2 (1.7)	
Education				
No formal, high school/GED, 2 years of college	84 (22.5)	210 (24.3)	43 (36.1)	0.00
Bachelor/masters	221 (59.2)	512 (59.3)	69 (58.0)	
Professional degree	68 (18.2)	142 (16.4)	7 (5.9)	
Income				
$0–49,999	108 (29.9)	294 (34.4)	48 (41.4)	0.16
$50,000–$99,999	147 (40.7)	346 (40.5)	41 (35.3)	
$100,000+	106 (29.4)	215 (25.1)	27 (23.3)	
Involvement in child’s schooling				
0–25%	59 (38.6)	138 (32.1)	15 (24.2)	0.40
25–49%	22 (14.4)	51 (11.9)	10 (16.1)	
50–74%	24 (15.7)	80 (18.6)	12 (19.4)	
75–100%	48 (31.4)	161 (37.4)	25 (40.3)	
Telehealth utilization during pandemic				
Yes	102 (27.6)	328 (38.4)	49 (41.2)	0.01
No	268 (72.4)	526 (61.6)	70 (58.8)	

^a^North = Massachusetts, New York, New Jersey, Pennsylvania, Ohio

^b^South = Virginia, North Carolina, South Carolina, Florida, Alabama, Louisiana

^c^West = California, Washington

*Significance was analyzed by Fisher’s exact test. Note: Significance was determined at α = 0.05

### Multinomial Logistic Regression

After controlling for the seven covariates in the multinomial logistics regression models, four predictors emerged for an increased risk of either moderate or severe levels of depression, anxiety, or stress. While we saw a significant difference in PHQ-9 levels and IES-R levels reported between healthcare and non-healthcare workers overall, after controlling for other covariates in logistic regression models, only type of healthcare worker emerged as a significant predictor of PHQ-9 level. The odds of reporting a severe PHQ-9 score among Nurses/Hospital Technicians were 7.92 times than reported by a doctor (Table 5). Gender was a significant risk factor for a moderate (OR 2.50, 95% CI 1.33–4.70) or severe (OR 10.17 with 95% CI 1.32–78.28) GAD-7 score and moderate IES-R (OR 3.26 with 95% CI 1.72–6.18) score among women as compared to men (Tables 6, 7). Significance was found when examining the GAD-7 score and ethnicity. Being Black/African American/Other appears to be protective against reporting a moderate GAD-7 score compared to White participants (Table 6). Finally, the odds that participants > 55 years of age are 0.40, (0.19–0.84) times the odds of those 18–34 years getting a moderate IES-R score, again indicating a protective effect (Table 7).

**Table 5:** Multinomial Logistic regression model of GAD-7 Score

Effect	GAD-7 Score	Odds ratio	95% confidence limits	
			Lower	Upper
Age				
35–54 vs 18–34	Moderate	1.41	0.82	2.44
35–54 vs 18–34	Severe	1.23	0.50	3.05
≥ 55 vs 18–34	Moderate	0.58	0.28	1.18
≥ 55 vs 18–34	Severe	0.56	0.16	1.98
Gender				
Female vs male	Moderate	2.50	1.33	4.70
Female vs male	Severe	10.17	1.32	78.28
Ethnicity				
Black/other vs white	Moderate	0.52	0.27	0.99
Black/other vs white	Severe	0.44	0.14	1.39
Healthcare provider role				
Nurse vs doctor	Moderate	1.41	0.58	3.43
Nurse vs doctor	Severe	1.05	0.24	4.63
Other vs doctor	Moderate	1.02	0.46	2.25
Other vs doctor	Severe	0.65	0.17	2.49
Marital status				
Married/domestic partnership vs single	Moderate	0.63	0.34	1.16
Married/domestic partnership vs single	Severe	0.89	0.32	2.50
Divorced/widowed/separated vs single	Moderate	0.68	0.31	1.52
Divorced/widowed/separated vs single	Severe	0.37	0.08	1.80
Education				
Bachelor/masters vs associate and under	Moderate	0.90	0.49	1.63
Bachelor/masters vs associate and under	Severe	0.51	0.19	1.36
Professional degree vs associate and under	Moderate	1.11	0.52	2.39
Professional degree vs associate and under	Severe	0.73	0.21	2.56
Income				
$50,000–$99,999 vs $0–49,999	Moderate	0.71	0.39	1.30
$50,000–$99,999 vs $0–49,999	Severe	0.54	0.20	1.47
$100,000+ vs $0–49,999	Moderate	0.68	0.32	1.42
$100,000+ vs $0–49,999	Severe	0.66	0.20	2.26

**Table 6:** Multinomial Logistic regression model of PHQ-9 Score

Effect	PHQ-9 Score	Odds ratio	95% confidence limits	
			Lower	Upper
Age				
35–54 vs 18–34	Moderate	1.39	0.82	2.36
35–54 vs 18–34	Severe	0.86	0.32	2.34
≥ 55 vs 18–34	Moderate	0.81	0.40	1.66
≥ 55 vs 18–34	Severe	0.89	0.26	3.01
Gender				
Female vs male	Moderate	1.66	0.88	3.12
Female vs male	Severe	4.01	0.51	31.47
Ethnicity				
Black/other vs white	Moderate	0.74	0.39	1.40
Black/other vs white	Severe	0.36	0.08	1.71
Healthcare provider role				
Nurse vs doctor	Moderate	1.34	0.56	3.19
Nurse vs doctor	Severe	7.92	1.41	44.44
Other vs doctor	Moderate	0.75	0.35	1.60
Other vs doctor	Severe	1.43	0.28	7.37
Marital status				
Married/domestic partnership vs single	Moderate	0.78	0.44	1.38
Married/domestic partnership vs single	Severe	0.83	0.28	2.51
Divorced/widowed/separated vs single	Moderate	0.92	0.42	1.99
Divorced/widowed/separated vs single	Severe	0.67	0.15	2.93
Education				
Bachelor/masters vs associate and under	Moderate	0.67	0.37	1.21
Bachelor/masters vs associate and under	Severe	0.55	0.21	1.45
Professional degree vs associate and under	Moderate	0.57	0.27	1.19
Professional degree vs associate and under	Severe	0.66	0.17	2.55
Income				
$50,000–$99,999 vs $0–49,999	Moderate	0.53	0.30	0.96
$50,000–$99,999 vs $0–49,999	Severe	0.37	0.13	1.04
$100,000+ vs $0–49,999	Moderate	0.53	0.26	1.09
$100,000+ vs $0–49,999	Severe	0.55	0.16	1.87

**Table 7:** Multinomial Logistic regression model of Impact of Event Scale Score

Effect	IES-R Score	Odds ratio	95% confidence limits	
			Lower	Upper
Age				
35–54 vs 18–34	Moderate	0.81	0.46	1.43
35–54 vs 18–34	Severe	0.84	0.28	2.57
≥ 55 vs 18–34	Moderate	0.40	0.19	0.84
≥ 55 vs 18–34	Severe	0.55	0.13	2.27
Gender				
Female vs male	Moderate	3.26	1.72	6.18
Female vs male	Severe	1.73	0.46	6.54
Ethnicity				
Black/other vs white	Moderate	0.78	0.40	1.52
Black/other vs white	Severe	0.47	0.10	2.23
Healthcare provider role				
Nurse vs doctor	Moderate	2.33	0.92	5.92
Nurse vs doctor	Severe	1.95	0.27	13.93
Other vs doctor	Moderate	1.96	0.85	4.52
Other vs doctor	Severe	0.88	0.13	5.77
Marital status				
Married/domestic partnership vs single	Moderate	0.93	0.50	1.73
Married/domestic partnership vs single	Severe	0.94	0.28	3.17
Divorced/widowed/separated vs single	Moderate	0.69	0.31	1.53
Divorced/widowed/separated vs single	Severe	0.61	0.12	3.01
Education				
Bachelor/masters vs associate and under	Moderate	0.86	0.46	1.59
Bachelor/masters vs associate and under	Severe	0.42	0.15	1.17
Professional degree vs associate and under	Moderate	1.35	0.61	3.01
Professional degree vs associate and under	Severe	0.30	0.06	1.59
Income				
$50,000–$99,999 vs $0–49,999	Moderate	1.00	0.54	1.85
$50,000–$99,999 vs $0–49,999	Severe	0.73	0.23	2.31
$100,000+ vs $0–49,999	Moderate	1.39	0.65	3.00
$100,000+ vs $0–49,999	Severe	1.24	0.30	5.07

## Discussion

As studies emerge showing who is most likely to be affected psychologically by an infectious disease outbreak, administrations and communities can begin to better prepare for dealing with these situations early and effectively. Understanding who is at risk will allow for better allocation of funds to mental health needs. The results of this study indicate that reported levels of depression, anxiety, and stress significantly differ across many variables, suggesting that different individual characteristics may be risk factors for experiencing mental health struggles during a global pandemic. Additionally, the results indicate that within a global pandemic, stress, anxiety, and depression are impacted in different ways. While we found that non-healthcare workers were more likely to experience moderate or severe depression, the results indicate that the role of a healthcare worker, specifically being a nurse or hospital technician presents, an increased risk for mental health outcomes. Researchers such as Pffeferbaum & North, (2020) pointed out how inadequate testing, limited treatment options, insufficient personal protective equipment and medical supplies, extended workloads, and other emerging concerns could be factors for psychological distress among healthcare providers. Studies also indicated that nurses are more likely to be affected by psychological distress due to infectious disease outbreaks (Douglas et al., 2009; Pffeferbaum & North, 2020; Bai et al., 2004; Greenberg et al., 2020; Shih et al., 2007). The results of our study align with the findings of these studies, as nurses had the highest cases of severe depression symptoms amongst their healthcare worker peers in the PHQ-9.

Our study showed that gender, ethnicity, and age also contribute to level of severity reported for the three mental health outcomes which indicated these personal factors should also be considered for targeting interventions and support. Interestingly, respondents who identified that they had a prior mental health diagnosis were at lower risk of psychological impact due to the COVID-19 pandemic. Our findings aligned with existing literature and the US Department of Veteran Affairs, who have shown that middle-aged adults, females, those with psychological disease who have gone untreated, and low socioeconomic status individuals are at greater risk for psychological impact due to a disaster or disease outbreak (Douglas et al., 2009; Lee et al., 2007). We also saw how individuals who received telehealth care during the pandemic were at lower risk of psychological distress. These findings emphasize the importance of online resources for individuals during times of crisis to help provide support and managed care for individuals at risk of psychological impact. These findings also align with research which has shown telehealth as “effective and adaptable solutions to the care of mental illnesses universally…especially in isolated communities” (Lanagarizadeh et al., 2017).

Often, stress and anxiety are the result of an underlying uncertainty or fear of something that is out of one's control. The COVID-19 pandemic quarantine and recommendations of social distancing have radically changed how our society functions. This is a first step towards understanding the impact of COVID-19 on mental health outcomes. While our results provide important findings for future research, the study has limitations that need to be considered. One major limitation to our study was that it was conducted under the setting of stay-at-home orders. We were constrained to surveying only individuals who had internet access and online social media accounts. This study employed a self-report survey methodology which is always subject to various forms of bias such as recall bias and self-report bias. While the sample size crossed 13 different states, the demographics were heavily skewed towards females, Non-Hispanic whites, and well-educated individual which was accounted for in the regression analysis. Even with strained generalizability, these results provide us insight into the impact of COVID-19 among this population on mental health outcomes, and implications for future support efforts during pandemics or times of crisis. Future research should continue to explore these topics among diverse populations in order to drawn stronger causal conclusions. Additionally, nationwide political uncertainties and social conflicts that emerged during our survey may have affected some responses and potentially heightened levels of distress.

As the pandemic continues to evolve, we have many lessons to learn and explore. Future studies should continue to explore additional factors playing a role in reported stress, anxiety, and depression levels, such as the role of social media in an individual’s mental wellbeing. Additional studies should explore the long-term effects of the COVID-19 pandemic on the psyche of healthcare providers.

In conclusion, our study has helped contribute to existing literature in better understanding the psychological impact of natural disasters and infectious disease outbreaks. Hopefully, these insights will lead to the provision of appropriate resources and treatments for those in need during times of crisis.
